# Measuring alcohol use among adolescents in Africa: A systematic scoping review of consumption, screening and assessment tools

**DOI:** 10.1111/dar.13715

**Published:** 2023-07-13

**Authors:** Maaike L. Seekles, Eleanor Briegal, Alice M. Biggane, Angela I. Obasi

**Affiliations:** ^1^ Department of International Public Health Liverpool School of Tropical Medicine Liverpool UK

**Keywords:** adolescent, Africa, alcohol, alcohol measurement tool, scoping review

## Abstract

**Issues:**

Globally, adolescent drinking is a major public health concern. Alcohol measurements are influenced by local consumption practices, patterns and perceptions of alcohol‐related harm. This is the first review to examine what tools are used to measure alcohol consumption, or screen for or assess harmful use in African adolescents, and how these tools take into account the local context.

**Approach:**

A systematic scoping review was conducted in line with the Arksey and O'Malley framework. A search in MEDLINE, CINAHL, Global Health and the Cochrane Database covered the period of 2000–2020.

**Key Findings:**

The search identified 121 papers across 25 African countries. A range of single‐ and multi‐item tools were identified. Very few adaptations of existing questions were specified, and this search identified no tools developed by local researchers that were fundamentally different from established tools often designed in the USA or Europe. Inconsistencies were found in the use of cut‐off scores; many studies used adult cut‐off scores.

**Implications and Conclusion:**

The possible impact of African drinking practices and culture on the accuracy of alcohol screening tools is currently unknown, but is also not taken into account by most research. This, in combination with a limited geographical distribution of alcohol‐related research across the continent and inconsistent use of age‐ and gender‐specific cut‐off scores, points towards probable inaccuracies in current data on adolescent alcohol use in Africa.

**Key Points Summary**

- This review identified no studies that specifically explored the applicability of existing alcohol measurement tools in African contexts.
- Adaptation of existing alcohol tools was mostly limited to translation.
- There is an apparent lack of studies on alcohol (mis)use in most African countries.
- Gender and/or age‐specific cut‐off scores for alcohol tools were applied inconsistently.

## 1 INTRODUCTION

Overconsumption of alcohol is a significant public health issue worldwide, with three million deaths per year attributable to alcohol use [1, 2, 3]. This is particularly pertinent in Africa, which has the highest burden of disease and injury attributed to alcohol of any world region [1]. Adolescent drinking in particular is a major global health concern [3] because of its negative impact on an adolescent's physical, academic, emotional and social development [4, 5]. Higher alcohol consumption in late adolescence continues into adulthood and is associated with alcohol problems, including dependence [6]. This makes adolescence a crucial time to intervene. To inform alcohol policy and identify those who might benefit from early intervention, measurement tools are needed that adequately capture adolescent drinking patterns and behaviours. Various such tools exist, which use indicators, defined as questions used to measure consumption (patterns) or screen for/assess harmful use or dependency [7]. Indicators of consumption levels include frequency and/or quantity questions, while those of harmful consumption patterns capture heavy episodic drinking (HED) or binge drinking. Consumption indicators can be used as single items or combined to gain insight into patterns, variability and magnitude of drinking. For example, the Timeline Follow Back (TLFB) tool asks participants to provide retrospective estimates of their daily drinking over a specified time period [8]. Multi‐item screening instruments like the CRAFFT (acronym for Car, Relax, Alone, Forget, Friends, Trouble) [9] and CAGE (acronym for Cut Down, Annoyed, Guilty and Eye Opener) [10] include dependency indicators capturing adverse consequences of drinking. The Alcohol Use Disorders Identification Test (AUDIT), one of the most widely used multi‐item tools, combines consumption indicators (frequency, quantity and HED) with dependency indicators [11]. Indicators are also often included in omnibus instruments, such as the Global School‐based Student Health Survey (GSHS), a collaborative surveillance project designed to help countries measure and assess a wide range of behavioural risk factors among young people aged 13–17 years [12].

Screening for alcohol misuse among adolescents presents specific challenges. Instruments need to take into consideration the age and developmental stage of adolescents, both in terms of question design and cut‐off scores used. This is particularly important for tools that assess HED. There currently is a lack of consensus about the standard definition of HED and the threshold at which it is achieved. The World Health Organization (WHO) suggests HED is reached when an adult (defined as 15+ years) consumes 60 g or more of pure alcohol in one occasion [13]. In the USA, the National Institute on Alcohol Abuse and Alcoholism recommends that the definition of HED is based on drinking behaviours that elevate an individual's blood alcohol concentration above the level of 0.08%. This corresponds to approximately five drinks for men, or four drinks for women, in 2 h [14]. Adolescents are likely to reach blood alcohol concentrations of more than 0.08% at lower levels of consumption due to factors such as smaller body size [15]. Donovan created theoretical adolescent thresholds for youth: in those aged 9–13, a HED episode was estimated to occur with intake of three or more drinks within a 2‐h period; for those aged 14–15, with four or more drinks for boys and three or more drinks for girls; and for those aged 16–17, with five or more drinks for boys and three or more drinks for girls [16].

Although the AUDIT was developed by a six‐country WHO collaboration (including Kenya), alcohol screening tools have mainly been developed and validated in the USA and European countries [7]. This does not guarantee validity in African countries. Measurements that rely on alcohol consumption data are highly influenced by local context, due to differences in standard serving sizes, alcohol content of beverages and the setting in which drinks are consumed (e.g., private or public place) across countries [13]. For example, in South Africa, where a standard drink is 12 mg of alcohol, HED is reached at five drinks (using the WHO threshold). Yet, in Tanzania, where a standard drink is 10 mg, the threshold would lie at six drinks. This then affects the phrasing and scoring of questions in well‐established tools such as the AUDIT, which was developed based on the assumption that one ‘drink’ contains approximately 10 g of alcohol. Its manual states that: ‘if in a particular culture, a typical drink contains more or less than 10 g alcohol, the questions should be rephrased or the response categories altered’ [11].

Accurate measurement of alcohol use among adolescents in Africa is particularly difficult in contexts where traditional homemade beverages (homebrew) represent the majority of alcohol use [17]. Measures for the consumption of homebrew are hard to standardise. This beverage is often shared from a large communal container and the strength of the drink may vary every time it is made. Local brew, as opposed to commercial drinks, is not always defined as alcohol. Furthermore, locals may quantify alcohol consumption in terms of money spent rather than the number of drinks consumed [17]. This raises issues, especially if tools that explore consumption based on questions related to quantity of drinks consumed do not specify what constitutes a standard drink. Locally appropriate monitoring tools are needed that reflect local alcohol consumption practices, patterns and perceptions of alcohol‐related harm. Particularly, tools that include dependency indicators (such as the AUDIT and CAGE) may be more sensitive to differences in linguistic interpretations and local manifestations and conceptualisations of alcohol problems than frequency, quantity and HED items. Therefore, cultural adaptation of international instruments goes beyond the correct translation of words. It should also explore the wider cultural aspects of alcohol consumption [13]. Not only does this aid cross‐national comparisons of consumption patterns, it might also ensure greater accuracy of screening tools used to determine whether a drinker is at risk of harm.

This scoping review aimed to systematically examine and map studies that captured alcohol consumption or risk of harm among adolescent populations in Africa. Specifically, it sought to explore how adolescent alcohol consumption is currently measured, which tools were used, and if and how such tools respond to the local context. A preliminary search conducted in MEDLINE and the Cochrane Database found no previous or underway systematic or scoping review on the topic. Providing a ‘state‐of‐the‐art’ overview, this review will inform researchers in their decisions around selecting/adapting measurement tools in their context. In addition, it will highlight areas for further research needed to advance the development/use of accurate, cross‐cultural adolescent alcohol tools.

## 2 METHODS

### 2.1 Overview

Since this review has a broad aim of informing researchers by examining how research on adolescent alcohol use in Africa is conducted (in terms of measurement tools used), the decision was made to undertake a scoping review, as opposed to a systematic review which typically addresses a narrow research question focused on informing practice [18]. This scoping review was conducted in accordance with the stepwise methodological framework described by Arksey and O'Malley [19], complemented by guidance from the Joanna Briggs Institute [20]. Study details were documented in advance in a protocol [21] which was registered on the Open Science Framework (https://osf.io/2q9su). This review is reported in accordance with the PRISMA Extension for Scoping Reviews guidelines [22].

### 2.2 Search strategy

We searched four electronic databases: MEDLINE, CINAHL, Global Health and the Cochrane Database of Systematic Reviews. The search strategy used terms relating to adolescents, Africa and alcohol consumption, screening and assessment tools. Search strategies can be found in Appendix S2, Supporting Information. The search terms were developed through an initial limited search of MEDLINE and CINAHL, with input from a health librarian specialist. All databases were searched on 9 July 2020, for entries from January 2000 to July 2020. Only papers published in English were considered for inclusion. Targeted Google searches were done to identify additional documents that were referenced in included papers. Five potentially relevant articles could not be accessed; attempts were made to contact the author or to access an interlibrary loan, however these were unsuccessful.

### 2.3 Inclusion criteria

Inclusion criteria have been developed using the population, concept and context framework.

#### 2.3.1 Population

While alcohol use among children younger than 10 occurs, is under‐researched and has a detrimental impact [23], we decided to focus this review on adolescence since this is a period characterised by a rapid increase of alcohol use. Papers that targeted adolescents, defined—in line with WHO guidance [24]—as individuals aged between 10 and 19 were included. However, many study samples were not defined by age, but rather by population group (e.g., secondary school students). This meant that in many studies, some participants were over the age of 19. To account for this variation, this scoping review included sources of information with participants up to the age of 30 if those in the 10–19 age group made up at least 50% of participants. Where only the mean or median age was reported, this was required to not be higher than 19 years.

#### 2.3.2 Concept

The core concept examined by this scoping review is alcohol measurement tools, including instruments for consumption, screening and assessment. Included studies reported data on adolescent alcohol consumption patterns or risk of harm. Studies only reporting lifetime use were excluded.

#### 2.3.3 Context

Articles that collected data from any country on the African continent were included.

### 2.4 Sources of evidence screening and selection

Following the search, all identified citations were imported into EndNoteX9 [25] reference management software and duplicates were removed. Following a pilot test of the first 500 results, title and abstract screening was done independently by two reviewers. Full‐text articles were also independently reviewed by two reviewers and disagreement about inclusion was resolved through discussion. In the event of no agreement being reached, a third member of the research team weighed in until a consensus was reached.

### 2.5 Data extraction and analysis

Data were extracted independently by two reviewers and added to a data charting table in Microsoft Excel. This was an iterative process and categories were added as familiarity with the literature increased. Extracted data included study type and year; country; population age and sex; context; setting; sample size; aim of measurement; type/name of alcohol tool; name of omnibus survey (if applicable); evidence of validation; cut‐off scores (if applicable); and responses to local context. For most categories, data were analysed descriptively, and a tabular overview was provided to aid a narrative summary. With regards to adaptations to the local context, data were analysed via content analysis and presented within themes.

## 3 RESULTS

### 3.1 Search results

**FIGURE 1:**
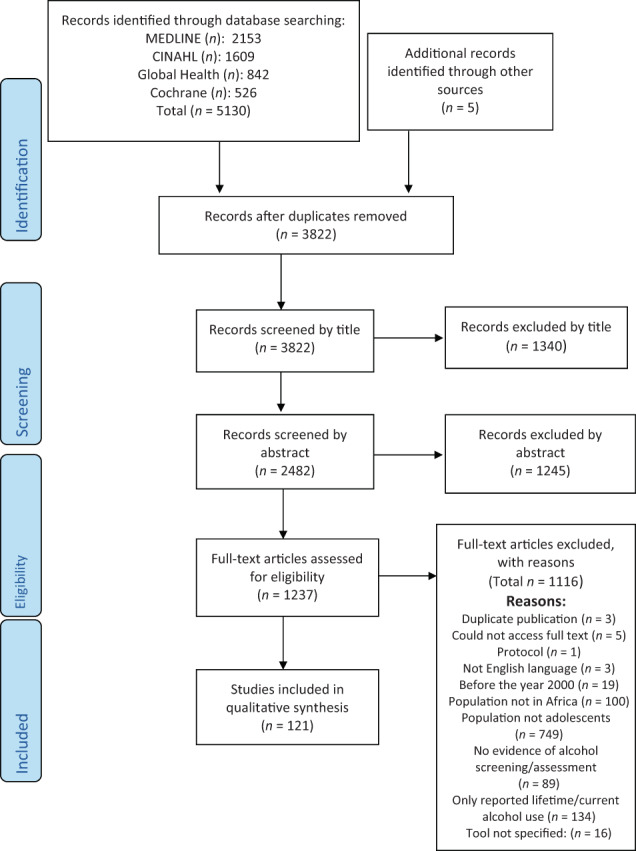
Preferred Reporting Items for Systematic Reviews and Meta‐Analyses (PRISMA) flow diagram.

The search yielded 5135 results (5130 from the original database search and 5 from other sources) of which 3822 remained after duplicates were removed. A further 1340 records were excluded by title and 1245 removed after abstract screening. The full texts of the remaining 1237 records were screened, and 1116 records were excluded. Specifically, 134 papers were excluded because they only reported data on lifetime or current alcohol use, as opposed to providing insight into drinking patterns or risk of harm. Two of these papers, both from the same lead author, reported the lifetime/current use of home‐brewed alcohol in South‐Africa, but did not capture consumption patterns/harm [26, 27] and were therefore excluded. A total of 121 papers were included in the final analysis (Figure 1).

### 3.2 Study characteristics and target populations

**FIGURE 2:**
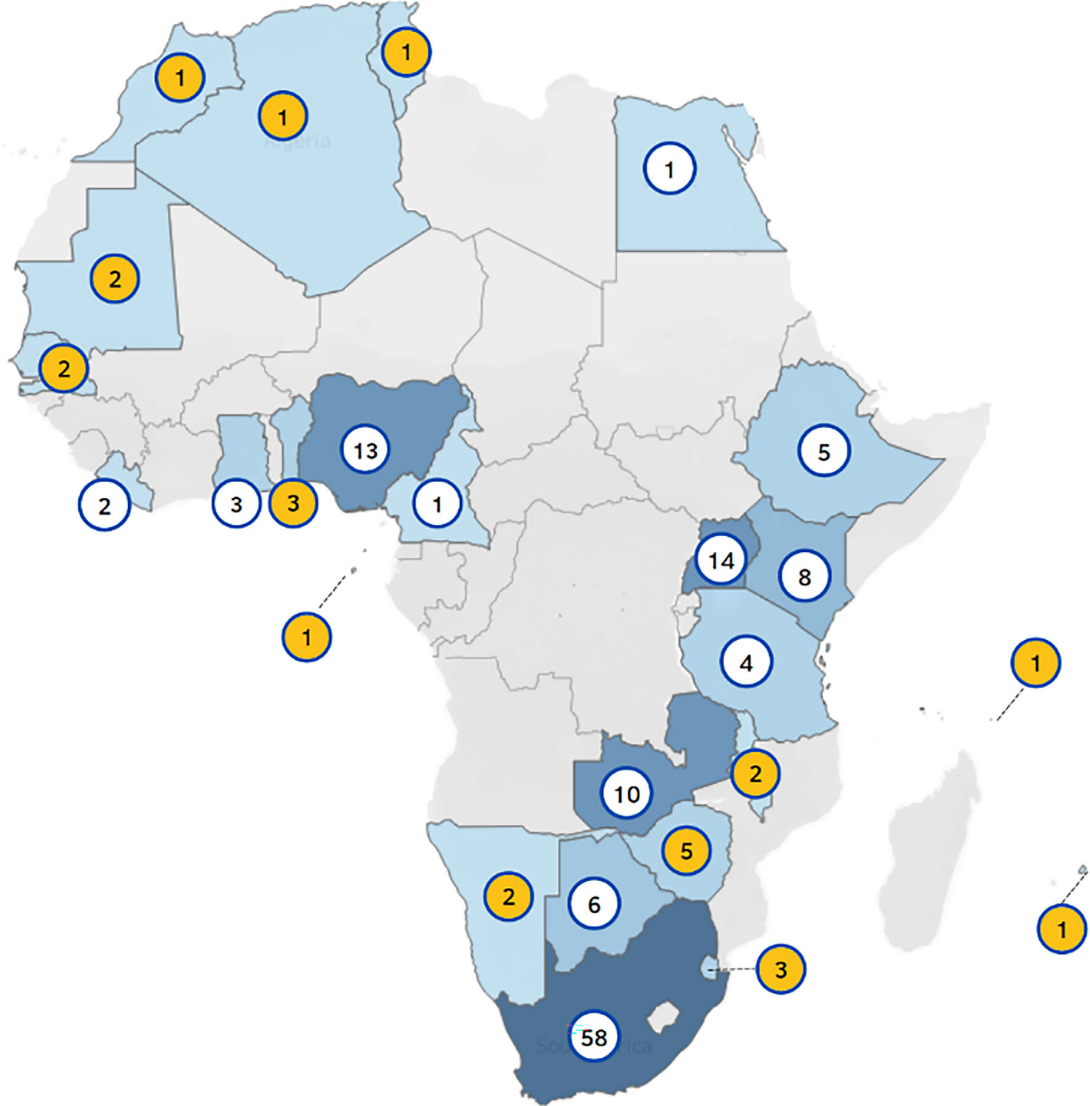
Overview of countries in which studies had taken place. *Note*: Several studies were multi‐country studies so have been represented more than once.

The search identified 95 cross‐sectional studies, 11 experimental (intervention) studies, 8 case‐cohort studies and 7 papers that explored psychometric properties of tools (including validation). Most descriptive or intervention studies aimed to measure alcohol consumption in the context of substance use (including smoking or drug use) or sexual health. Few descriptive studies (*n* = 15, 14.6%) aimed to exclusively capture alcohol consumption and only one intervention solely aimed to reduce alcohol use. Instead, studies captured alcohol use as an associative factor related to other (sexual) risky behaviours or violence. As seen in Figure 1, studies presented data from 25 African countries, with most studies undertaken in South Africa (*n* = 58), Uganda (*n* = 14) or Nigeria (*n* = 13). Notably, all intervention studies were conducted in South Africa. In 13 countries (yellow circles Figure 2), data on alcohol consumption were exclusively collected through the GSHS, as opposed to local research initiatives.

Only one study [28] included participants across the whole range of adolescence as defined by the WHO (10–19 years). Participant age ranges were not reported in 17 studies, which only reported mean or median age. Around half of descriptive studies included young adolescents (10–14 years); around 45% of intervention studies focused exclusively on young people aged 18 years and over. Most studies presented data from both sexes and took place in a school or community setting (Table 1).

**TABLE 1:** Context, setting and population of studies.

Characteristic	Descriptive studies, *n* (%)	Intervention studies, *n* (%)
Total	103	11
Context		
Substance use	27 (26.2)	4 (36.4)
Sexual health	26 (25.2)	5 (45.5)
Mental health	17 (16.5)	‐
Alcohol use	15 (14.6)	1 (9.1)
Health risk behaviour	9 (8.7)	‐
Development (brain, skeletal)	6 (5.8)	‐
Weight management	1 (1.0)	1 (9.1)
Bullying	1 (1.0)	‐
Violence	1 (1.0)	‐
Setting		
School (primary, secondary)	50 (48.5)	4 (36.4)
Community	27 (26.2)	3 (27.3)
University	13 (12.6)	1 (9.1)
Medical institution	11 (10.7)	3 (27.3)
Correctional institution	2 (1.9)	‐
Adolescent age range		
≤14 years	55 (53.4)	3 (27.3)
≤17 years	80 (77.7)	6 (54.5)
18–25 years (young adults)	6 (5.8)	5 (45.5)
Only mean/median provided	17 (16.5)	‐
Sex		
Both	95 (92.2)	8 (72.3)
Female	6 (5.8)	2 (18.2)
Male	2 (1.9)	1 (9.1)

### 3.3 Alcohol consumption, screening and assessment tools used

Table 2 provides an overview of relevant data that were extracted from the 114 descriptive and intervention papers. These were divided into four categories, based on the purpose of use of the tool as either measuring (consumption/behaviours), screening or assessing: tools were either used to simply report consumption patterns (Category 1); capture potentially harmful behaviours (Category 2); screen to identify those at risk of harm (using cut‐off scores, Category 3); or use an interview to assess psychopathology (Category 4).

**TABLE 2:** Studies categorised by purpose and type of alcohol‐related data collection.

Author (year)	Country	Tool	Cut‐off	HED threshold	Consumption or dependency	Part of named survey
Category 1: Reporting of consumption: No threshold/cut‐off applied—no reporting of prevalence of those at risk						
Arega (2019)	Ethiopia	Frequency item	N/A	N/A	Consumption	
Bekele (2011)	Ethiopia	Frequency item	N/A	N/A	Consumption	
De Santiago (2020)	São Tomé & Príncipe	Frequency item	N/A	N/A	Consumption	
D'Souza (2011)	South Africa	Quantity item	N/A	N/A	Consumption	
Eze (2017)	Nigeria	ADCQSSS	N/A	N/A	Consumption	
Getachew (2019)	Ethiopia	Frequency item Quantity item	N/A	N/A	Consumption	
Habesha (2015)	Ethiopia	Frequency item	N/A	N/A	Consumption	
Harris (2012)	Liberia	Frequency item	N/A	N/A	Consumption	
Iorfa (2018)	Nigeria	Frequency item	Not reported	N/A	Consumption	Psychoactive substance use (Eze, 2006) (not found)
Karnell (2006)	South Africa	Frequency item Quantity item Problem scale	N/A	N/A	Change in: Both	
Lentiro (2019)	Ethiopia	Frequency item	N/A	N/A	Consumption	
McKinnon (2016)	Multi‐country	Frequency item	N/A	N/A	Consumption	GSHS
Mojorele (2006)	South‐Africa	Frequency item	N/A	N/A	Consumption	
Motamedi (2016)	South Africa	Quantity item	N/A	N/A	Consumption	
Mpofu (2006)	South‐Africa	Quantity item	N/A	N/A	Consumption	SA Youth Health Survey
Ndetei (2010)	Kenya	Quantity item	N/A	N/A	Consumption	
Ochen (2019)	Uganda	Frequency item	N/A	N/A	Consumption	
Okigbo (2014)	Liberia	Frequency item	N/A	N/A	Consumption	
Oshodi (2010)	Nigeria	Frequency item	N/A	N/A	Consumption	WHO Student drug use quest
Refaat (2004)	Egypt	Frequency item	N/A	N/A	Consumption	
Steyl (2011)	South Africa	Quantity item	N/A	N/A	Consumption	
Taylor (2003)	South Africa	Frequency item	N/A	N/A	Consumption	
Vorster (2019)	South Africa	Frequency item Quantity item	N/A	N/A	Consumption	
Yongshi (2019)	South Africa	Frequency item	N/A	N/A	Consumption	
Category 2: Proportion of those with problematic behaviour, identified through positive answer to single‐item tool (no threshold/cut‐off)						
Leung (2019)	Multi‐country	Frequency item Intoxication item Problem item	N/A	N/A	Both	GSHS
Peltzer (2010a)	Multi‐country	Intoxication item Problem item	N/A	N/A	Both	GSHS
Swahn (2011)	Zambia	Frequency item Intoxication item Problem item	N/A	N/A	Both	GSHS
Morakinyo (2003)	Nigeria	Pimrat‐Awele drug quest.	N/A	N/A	Both	N/A
Peltzer (2010b)	Multi‐country	Intoxication item	N/A	N/A	Consumption	GSHS
Peltzer (2010c)	Botswana	Intoxication item	N/A	N/A	Consumption	GSHS
Doku (2011)	Ghana	Freq. of intoxication	N/A	N/A	Consumption	
Kagimu (2012)	Uganda	Intoxication item	N/A	N/A	Consumption	
Mataure (2002)	Zimbabwe	Frequency item + Intoxication item	N/A	N/A	Consumption	
Meghdadpour (2012)	South Africa	Frequency item + Intoxication item	N/A	N/A	Consumption	
Muula (2007)	Zambia	Freq. of intoxication	N/A	N/A	Consumption	GSHS
Muula (2013)	Zambia	Frequency of intoxication	N/A	N/A	Consumption	GSHS
Ndetei (2009)	Kenya	Frequency item + Intoxication item	N/A	N/A	Consumption	The School Toolkit
Parry (2004)	South‐Africa	Frequency item Freq. of intoxication	N/A	N/A	Consumption	
Randall (2014)	Benin	Freq. of intoxication	N/A	N/A	Consumption	GSHS
Rowland (2010)	South Africa	Freq. of intoxication	N/A	N/A	Consumption	
Rudatsikira (2007)	Kenya	Frequency item Freq. of intoxication	N/A	N/A	Consumption	GSHS
Shayo (2019)	Tanzania	Freq. of intoxication	N/A	N/A	Consumption	GSHS
Siziya (2008)	Zambia	Frequency item + Freq. of intoxication	N/A	N/A	Consumption	GSHS
Sumner (2016)	Kenya	Frequency item + Freq. of intoxication	N/A	N/A	Consumption	Violence Against Children Survey
Swahn (2012)	Uganda	Intoxication item	N/A	N/A	Consumption	
Dietrich (2013)	South Africa	Passing out item	N/A	Once in 6 months	Consumption	
Category 3: Screening for risky drinking by applying threshold/cut‐off						
Ibe (2011)	Nigeria	Frequency item	Taking ≥1 drink daily	N/A	Consumption	No
Yang (2019)	Zambia	Frequency item	Consuming ≥3 times a week	N/A	Consumption	
Cluver (2015)	South Africa	Frequency item	Consuming daily or several times a week	N/A	Consumption	
Peltzer (2009)	Multi‐country	Frequency item + Quantity item	Having ≥2 drinks on 20 days or more.	N/A	Consumption	GSHS
Chekib (2017)	Tunesia	FACE	Not specified	N/A	Both	
Chaiyasong (2018)	South‐Africa	International Alcohol Control Study survey, incl. RAPS4	‘Lower’: ≤4 drinks/occasion or 4–6 drinks/occasion < once per week; ‘increased’: 4–6 drinks/occasion ≥ once a week or 6+ drinks/occasion < once a week ‘high’: > 6 drinks/occasion ≥ once a week	N/A	Both	
Auerbach (2018a)	South‐Africa	AUDIT	≥16 OR a score 8–15 with ≥4 on dependency q's	Q3 (HED): not defined	Both	WHO WMHICSI
Auerbach (2018b)	South‐Africa	AUDIT	≥8 OR a score 8–15 with ≥4 on dependency q's	Q3 (HED): nd	Both	WHO‐WMHICSI
Ebert (2019)	South Africa	AUDIT	≥8 OR a score 8–15 with ≥4 on dependency q's	Q3 (HED): nd	Both	WHO‐WMHICSI
Olley (2008)	Nigeria	AUDIT	≥9 hazardous ≥19 harmful	Q3: ≥5 drinks	Both	
Jewkes (2010)	South Africa	AUDIT	≥8 hazardous	Q3 (HED): nd	Both	Stepping Stones Study
Jina (2012)	South Africa	AUDIT	≥8 hazardous	Q3 (HED): nd	Both	Stepping Stones Study
Adams (2013)	South Africa	AUDIT	≥8 hazardous ≥13 women/≥15 men: dependence	Q3 (HED): nd	Both	
Nduna (2013)	South Africa	AUDIT	≥8 hazardous	Q3 (HED): nd	Both	Stepping Stones Study
Kaufman (2014)	South Africa	AUDIT	≥8 hazardous ≥15 harmful	Q3 (HED): nd	Both	
Martin (2014)	South Africa	AUDIT	≥8 hazardous	Q3 (HED): nd	Both	
Francis (2015)	Tanzania	AUDIT + TLFB	≥8 hazardous	Q3: ≥6 drinks	Both	
Mabaso (2018)	South Africa	AUDIT	≥8 high‐risk ≥20 hazardous	Q3 (HED): nd	Both	SA National HIV Prevalence, Incidence, Behaviour Survey
Riva (2018)	Botswana	AUDIT	≥5 hazardous	Q3 (HED): nd	Both	
Hogarth (2019)	South Africa	AUDIT	≥8 hazardous	Q3 (HED): nd	Both	
Maserumule (2019)	South Africa	AUDIT	≥8 hazardous ≥13 women/≥15 men: dependence	Q3 (HED): nd	Both	
Pengpid (2013)	South Africa	AUDIT	≥8 problematic 9–19 hazardous 20–40 dependence	Of ≥4 women, ≥5 for men on one occasion	Both	
Rotheram‐Borus (2013)	South Africa	AUDIT	≥9 hazardous	≥6 drinks in a day	Both	
DeAtley (2020)	South Africa	AUDIT‐C	Not used (overall score reported)	≥3 drinks for girls/≥4 for boys	Consumption	
Chirinda (2014)	South‐Africa	AUDIT‐C	AUDIT‐C ≥ 5	≥5 drinks/occasion	Consumption	
Matshipi (2019)	South‐Africa	Frequency item HED item CAGE	CAGE: ≥2	≥4 bottles of 750 mL for girls, ≥5 for boys, or 1 L of communal drink	Both	
Swahn (2018)	Uganda	Frequency item Quantity item Freq. of intoxication HED item Problem items CAGE	– – CAGE: ≥2	≥5 drinks in one day ≥3 drinks on a typical day	Both	Kampala Youth Survey
Swahn (2019)	Uganda	Frequency item Quantity item Freq. of intoxication HED item Problem items CAGE	CAGE ≥2	≥5 drinks in one day	Both	Kampala Youth Survey
Swahn (2020)	Uganda	Frequency item Quantity item Freq. of intoxication HED item Problem items CAGE	CAGE ≥2	≥5 drinks in one day	Both	Kampala Youth Survey
Culbreth (2020)	Uganda	CAGE	CAGE ≥2	N/A	Dependency	Kampala Youth Survey
Peer (2013)	South Africa	CAGE	CAGE ≥2	N/A	Dependency	SA Demographic & Health
Dada (2016)	Nigeria	Frequency item Freq. of intoxication CAGE	CAGE ≥2	N/A	Both	N/A
Stevanovic (2015)	Nigeria	CRAFFT	Not used	N/A	Dependency	
Atitola (2013)	Nigeria	CRAFFT	CRAFFT ≥2	N/A	Dependency	
Atitola (2017)	Nigeria	CRAFFT	CRAFFT >2	N/A	Dependency	N/A
Moodley (2014)	South‐Africa	CRAFFT HED item	CRAFFT ≥2	≥5 drinks/single day	Both	
Chauke (2015)	South‐Africa	Student alcohol questionnaire (problem + HED)	N/A	>6 drinks/occasion	Both	
Okike (2020)	Nigeria	Quantity item		≥4 bottles a day	Consumption	
Ayo‐Yusuf (2009)	South‐Africa	HED item	N/A	≥5 drinks in a row	Consumption	No
Ayo‐Yusuf (2013)	South‐Africa	HED item	N/A	≥5 drinks in a row	Consumption	No
Carney (2019)	South Africa	HED item	N/A	≥4 drinks/occasion	Consumption	Rev Risk Behaviour Assessment
Ghuman (2015)	South‐Africa	Frequency item HED item	N/A	Not defined	Consumption	No
Harker (2020)	South Africa	International Alcohol Control Survey (RAPS4)	RAPS4: ≥1	≥6 drinks females; ≥8 males/occasion	Both	No
Jemmott (2019)	South Africa	HED item	N/A	≥5 drinks/occasion	Consumption	
Jonas (2016)	South‐Africa	HED item	N/A	≥5 drinks/occasion	Consumption	YRBS
Mertens (2014)	South Africa	HED item ASSIST	ASSIST: ≥11	≥3 female or ≥6 male/occasion	Both	
Myers (2019)	South Africa	HED item	N/A	≥4/occasion	Consumption	
O'Leary (2015)	South‐Africa	HED item	N/A	≥5 drinks/occasion	Consumption	No
Peitzmeier (2016)	South‐Africa	HED item	N/A	≥5 drinks in a row	Consumption	No
Ramsoomar (2012)	South‐Africa	HED item	N/A	≥5 drinks within a few hours on 1 or more days	Consumption	SADHS + YRBS + NIMSS
Reddy (2007)	South‐Africa	Frequency item HED item	N/A	≥5 drinks/few hours on 1 or more days	Consumption	YRBS
Visser (2017)	South Africa	HED item		≥5 drinks/occasion	Consumption	
Oduniaya (2015)	South Africa	HED item		≥6 drinks/typical day	Consumption	
Mehra (2014)	Uganda	Frequency item HED item		≥6 drinks	Consumption	
Oppong Asante (2015)	Ghana	Frequency item		Drinking 3 times or more in a week	Consumption	
Porter (2009)	South‐Africa	HED item Frequency item	N/A	≥5 drinks/occasion. Alcohol at least 20/30 days/month	Consumption	Risk behaviour survey
Ward (2015; intervention)	South Africa	HED item ASSIST	ASSIST cut‐off not specified	≥3 (women) ≥5 (men)/occasion	Both	
Category 4: Psychopathology assessed through interview						
Atitola (2012)	Nigeria	K‐SADS‐PL	N/A		Both	N/A
Carney (2020; intervention)	South Africa	Adolescent Diagnostic Interview TLFB Frequency item	N/A		Both	
Ferret (2010)	South‐Africa	K‐SADS‐PL SSAGA‐II L TLFB	Lifetime use of 100 units (TLFB)		Both	N/A
Ferret (2011)	South‐Africa	K‐SADS‐PL TLFB	Lifetime use of 100 units (TLFB)		Both	N/A
Fein (2013)	South‐Africa	K‐SADS‐PL TLFB	Lifetime use of 100 units (TLFB)		Both	N/A
Brooks (2014)	South Africa	K‐SADS‐PL TLFB	Lifetime use of 100 units (TLFB)		Both	N/A
Dalvie (2014)	South‐Africa	K‐SADS‐PL TLFB	Lifetime use of 100 units (TLFB)		Both	N/A
Naude (2011)	South Africa	K‐SADS‐PL SSAGA‐II TLFB	Lifetime use of 100 units (TLFB)		Both	N/A
Naude (2011b)	South Africa	K‐SADS‐PL SSAGA‐II TLFB	Lifetime use of 100 units (TLFB)		Both	N/A
Naude (2012)	South Africa	K‐SADS‐PL SSAGA‐II TLFB	Lifetime use of 100 units (TLFB)		Both	N/A
Khasakhala (2013)	Kenya	MINI KID	N/A		Both	N/A

Abbreviations: ADCQSSS, Alcoholic Drinks Consumption Questionnaire for Secondary School Students; ASSIST, Alcohol, Smoking and Substance Involvement Screening Test; AUDIT, Alcohol Use Disorders Identification Test; AUDIT‐C, Alcohol Use Disorders Identification Test‐Consumption; CAGE, Cut Down, Annoyed, Guilty and Eye Opener; CRAFFT, Car, Relax, Alone, Forget, Friends, Trouble; FACE, fast alcohol consumption evaluation; GSHS, Global School‐based Student Health Survey; HED, heavy episodic drinking; K‐SADS‐PL, Kiddie Schedule for Affective Disorders and Schizophrenia, Present and Lifetime version; MINI KID, Mini International Neuropsychiatric Interview for Children and Adolescents; N/A, not applicable; nd, not defined; NIMSS, National Injury and Mortality Surveillance Study; RAPS4, Rapid Alcohol Problem Screen 4; SADHS, South African Demographic and Health Survey; SSAGA, Semi‐Structured Assessment for the Genetics of Alcoholism; TLFB, Timeline Follow Back; WMHICSI, World Mental Health International College Student Initiative; WHO, World Health Organization; YRBS, Youth Risk Behaviour Study.

Category 1 included 24 papers (21%, Table 2). The authors of these papers presented data on consumption patterns, without classifying participants as ‘at risk’. They mostly used a single‐item tool to present data on frequency or quantity of alcohol use. In four of these studies, the frequency/quantity item(s) were part of a named substance use questionnaire (WHO Student Drug Use Questionnaire [29] and Psychoactive Substance Use Questionnaire [30]) or an omnibus risk behaviour survey (GSHS [31] and the South Africa Youth Health survey [32]). Eze et al. had developed a multi‐item frequency tool, the Alcoholic Drinks Consumption Questionnaire for Secondary School Students [33]. One intervention study [34] reported a change in the number of alcohol‐related problems, captured by a ‘problem scale’. A reference search for this scale returned no results.

The authors of papers in Category 2 (*n* = 22, 19%) presented data on what proportion of their participants had displayed behaviours or experienced alcohol‐related problems that could indicate a risk of harm. Responses were usually captured on a Likert scale, which authors typically transposed into binary variables to present proportions of the population at risk (e.g., those who experienced drunkenness once or more were grouped together). Most authors used one or more of the following single items: frequency of intoxication (*n* = 10); lifetime/current intoxication (*n* = 8); problems (*n* = 3) and/or passing out (*n* = 1). In 14 papers, these single items were part of a reliable omnibus survey, namely the GSHS (*n* = 11 [35, 36, 37, 38, 39, 40, 41, 42, 43, 44, 45]), School Toolkit (*n* = 1, [46]) or the Violence Against Children Survey (*n* = 1 [47]). One study used a multi‐item tool, the ‘Pimrat‐Awele drug use questionnaire’ [48]. Although it was stated that the tool was validated, no evidence of this was found.

The authors of papers in Category 3 (*n* = 57, 50%,) screened for possible harmful drinking/dependency by applying a threshold to survey responses. This includes papers that estimated the prevalence of HED with a single HED item. In around half of the papers in this category (*n* = 30), authors screened for harm by combining questions related to consumption patterns and consequences of drinking. Authors of five papers only explored problematic consequences of drinking. Authors of 23 papers only explored consumption patterns, many of which (*n* = 16) used one single item (quantity, frequency or HED) as a screener. Multi‐item screening tools that have been validated (although some only to a limited extent in adolescents), were used in 35 studies. The AUDIT, which has the most validation evidence [7], was the most frequently used multi‐item tool (*n* = 18). Other tools that were used include the AUDIT‐C (consumption only), the CRAFFT, CAGE, Rapid Alcohol Problem Screen 4 (consequences only) and the Alcohol, Smoking and Substance Involvement Screening Test (ASSIST; consumption and consequences). Some studies used broader surveys, such as the Kampala Youth Survey [49, 50] and the International Alcohol Survey [51], in which some of these validated tools were incorporated. Chekib et al. used the Fast Alcohol Consumption Evaluation tool to determine the prevalence of ‘strong addiction’ [52], which combines questions of validated tools, including the AUDIT and CAGE. While good psychometric properties of this tool against the AUDIT were reported in French adults [53], evidence of validation in adolescents, or in an African setting, was not found.

Category 4 included 11 papers (10%). The authors of these papers used an interview (K‐SADS‐PL, Mini International Neuropsychiatric Interview for Children and Adolescents or Semi‐Structured Assessment for the Genetics of Alcoholism) as an assessment tool to diagnose participants with alcohol use disorder based on the Diagnostic and Statistical Manual of Mental Disorders criteria. Eight of these papers were from the same research group at the University of Cape Town, who used a similar methodology in their studies to better understand the effects of alcohol use on the adolescent brain, biochemical immunological parameters and skeletal development. They used the K‐SADS‐PL to determine whether participants fit the inclusion criteria in their study and used TLFB to capture consumption patterns; four of these studies then also used the Semi‐Structured Assessment for the Genetics of Alcoholism interview.

Table 3 provides an overview of the alcohol screening and assessment tools used. Most authors used (a combination of) single‐item tools. The authors of 56% of papers (*n* = 64) used consumption tools only; 4% (*n* = 5) used dependency tools only; and 39% (*n* = 45) used both. Validated, multi‐item screening and assessment tools were used in 39% (*n* = 44) of papers. Apart from in two validation studies (below), none of the authors used biomarkers, for example, phosphatidylethanol.

**TABLE 3:** Overview of identified alcohol consumption, screening or assessment tools. N.B. some papers used a combination of items, therefore the total percentage does not amount to 100%.

Tool used	Consumption or dependency	Descriptive studies (*n* = 103)	Intervention studies (*n* = 11)
		*n* (%)	*n* (%)
Single‐item tool			
Frequency: e.g., On how many days have you had an drink in the past month?	Consumption	39 (37.9)	2 (18.2)
HED: e.g., How often during the past 30 days did you have 5 or more drinks of alcohol in a row?	Consumption	17 (16.5)	5 (45.5)
Frequency of intoxication: e.g., During your life, how often did you drink so much alcohol that you were really drunk/ you passed out?	Consumption	13 (12.7)	
Intoxication (lifetime/current): Have you ever been drunk? (Mostly from GSHS)	Consumption	10 (9.7)	
Quantity: e.g., How many drinks do you drink on a typical day/last month?	Consumption	9 (8.7)	3 (27.3)
Problems: During your life, how many times have you ever had a hangover, felt sick, got into trouble with your family or friends, missed school, or got into fights, as a result of drinking alcohol (GSHS)?	Consumption	5 (4.9)	
Named/referenced multi‐item tool			
Alcoholic Drinks Consumption Questionnaire for Secondary School Students (Frequency item)	Consumption	1 (1.0)	
Student alcohol questionnaire (Frequency + HED item)	Consumption	1 (1.0)	
Pimrat‐Awele Substance use Questionnaire	Both	1 (1.0)	
International alcohol control study survey (incl. frequency, quantity, RAPS4)	Both	2 (1.9)	
FACE	Both	1 (1.0)	
Problem scale (Mitic et al., reference not found)	Dependency		1 (9.1)
Validated multi‐item screening tool			
AUDIT	Both	16 (15.5)	2 (18.2)
TLFB	Consumption	10 (9.7)	1 (9.1)
CAGE	Dependency	6 (5.8)	
CRAFFT	Dependency	4 (3.9)	
AUDIT‐C	Consumption	2 (1.9)	
ASSIST	Both		2 (18.2)
Diagnostic assessment tool			
K‐SADS‐PL (interview)	Both	9 (8.7)	
SSAGA‐II (interview)	Both	4 (3.9)	
MINI‐KID (interview)	Both	1 (1.0)	
Adolescent Diagnostic Interview	Both		1 (9.1)

Abbreviations: ASSIST, Alcohol, Smoking and Substance Involvement Screening Test; AUDIT, Alcohol Use Disorders Identification Test; AUDIT‐C, Alcohol Use Disorders Identification Test‐Consumption; CAGE, Cut Down, Annoyed, Guilty and Eye Opener; CRAFFT, Car, Relax, Alone, Forget, Friends, Trouble; FACE, fast alcohol consumption evaluation; GSHS, Global School‐based Student Health Survey; HED, heavy episodic drinking; K‐SADS‐PL, Kiddie Schedule for Affective Disorders and Schizophrenia, Present and Lifetime version; MINI KID, Mini International Neuropsychiatric Interview for Children and Adolescents; RAPS4, Rapid Alcohol Problem Screen 4; SSAGA, Semi‐Structured Assessment for the Genetics of Alcoholism; TLFB, Timeline Follow Back.

### 3.4 Use of cut‐off scores

Table 2 also provides an overview of the cut‐off scores that were used to determine risk of harm. Data on HED were collected in 46 papers (40.3%), mostly through a single HED item or as part of a larger questionnaire, such as the AUDIT. The threshold at which HED was reached was not specified by authors of 14 papers, most of whom used the AUDIT. Researchers of six papers used a lower HED threshold for females than for males. In most papers (*n* = 16), researchers applied a threshold of five or more drinks, yet this varied from as low as three or more drinks (for females) to as high as eight or more drinks (for males) in other studies. Oppong Asante et al. used a single frequency item to determine HED, with people drinking more than three times a week classed as heavy episodic drinkers [54]. Similar inconsistencies in use of cut‐off scores were found for other tools. For the AUDIT, most researchers used a score of eight or more as a lower‐level cut‐off for potential hazardous use, yet, some used more than eight, nine or more, or even 16 or more. Cut‐off scores to determine higher‐level problems, such as potential dependence, varied more widely across papers, and not all researchers used these scores (Table 2). In two papers, a lower score for females than for males was applied. Riva et al. were the only ones to report the use of a lower cut‐off score of five or more, citing that this was adolescent appropriate [55]. Cut‐off scores for the CRAFFT varied from 2 or more, to more than 2. The variation in these scores came from two papers written by the same author in different years [56, 57].

### 3.5 Evidence of adaptation to the local context

Of all the papers that explored consumption (*n* = 109), only six specifically asked respondents about the use of local beverages. Francis et al. [58], when asking about the consumption of local drinks, used a pictorial display of locally available beers to clarify the number of standard drinks in those beers. Matshipi et al. [59] measured one drink as either one bottled drink (with names of locally available bottles provided), or the communal drinking of a 1 L size container. Eze et al. asked students to list their consumption of local spirits, local cocktails and palm wine [33]. Swahn et al., in the Kampala Youth Survey [49], asked about the use of local brews and spirits, but did not link this to a quantity question. Finally, two studies reported results of the South‐African arm of the International Alcohol Control study [51, 60], which aims to collect comparable measures of alcohol consumption across countries. The survey uses a ‘within‐location beverage‐specific framework’, which first asks about the typical frequency of drinking in all locations where drinking occurs. Respondents then report their consumption of different beverages specific to their country, including informal alcohol, in their own terms. This is then coded by interviewers, which means that respondents do not have to ‘calculate’ and report their consumption in terms of standard drinks [61].

In terms of linguistic adaptations, two studies used adapted screening tools. Atilola et al. changed the word ‘car’ in the CRAFFT to ‘car/motorbike’ to reflect the frequent use of motorbikes in Nigeria (for question: have you ever driven in a car driven by someone—including yourself—who had been using alcohol) [56]. In South Africa, Karnell et al. adapted their problem scale by changing the word ‘party’ into ‘bash’ after participants explained that alcohol was only consumed in large quantities at the latter [34]. Matshipi et al. stated that there were no disparities between the original English CAGE and their Setswana version [59]. Although Auerbach et al. stated that their ‘translation and harmonisation process followed a protocol to maximise cross‐national equivalence of meaning and consistency of measurement’ [62, p. 5], no detail was provided into any adaptations made. Adaptations of assessment tools were not specified and there were no papers that specifically explored linguistic/cultural appropriateness or understanding of any tool. None of the papers examined how alcohol use was shaped by wider cultural factors, such as social norms and stigma. As a different type of adaptation, Mertens et al. responded to high prevalence rates of unplanned pregnancy and foetal alcohol syndrome in their South‐African study setting by lowering the threshold for binge drinking for female participants [63].

### 3.6 Explorations of psychometric properties

Five studies explored the validity of alcohol screening tools: the AUDIT (Uganda) [64] and CRAFFT (Nigeria) [65] were found to be valid, with good diagnostic properties against the Diagnostic and Statistical Manual of Mental Disorders assessment tools and/or a biomarker. The ASSIST (Zambia) was also found to be reliable and valid as a self‐report tool [66]. The MINI (Tanzania) [67] and AUDIT‐C (South‐Africa) [68] performed unsatisfactorily. Two further studies explored the reliability of two omnibus risk behaviour surveys, the Communities That Care Youth Survey (South Africa) [69] and the Kilifi Health Risk Behaviour Survey (Kenya) [70]. Both surveys included questions to capture data on alcohol consumption patterns and were found to be reliable.

## 4 DISCUSSION

The aim of this scoping review, covering the period 2000–2020, was to map research on adolescent alcohol use and specifically explore the use of alcohol consumption, screening and assessment tools among adolescent populations in Africa. It identified eight unique single‐item tools, which were often embedded in larger surveys such as the GSHS. In addition, eight multi‐item screening tools were identified, including the AUDIT, AUDIT‐C, ASSIST, CAGE, CRAFFT, Rapid Alcohol Problem Screen 4 and TLFB. The use of biomarkers in African adolescents is currently negligible, potentially due to the need for laboratory facilities to analyse samples, with phosphatidylethanol used in only two validation studies. The results identified no tools that consisted of questions that were substantially different from tools that are well established in European or North‐American contexts.

In terms of assessing levels of alcohol consumption, it was rare for papers to explore the consumption of local drinks, or to add a description of what constitutes a standard drink with specific reference to locally available alcohol. Furthermore, an explanation of how the size of a standard drink affects the phrasing or scoring of quantity or HED questions was usually lacking. One survey, that of the International Alcohol Control Study [51], was specifically designed to also capture informal and communal drinking of alcohol. Although the focus of this study was not on adolescents specifically, it may offer important lessons about measuring alcohol consumption in varying contexts and locations.

The accuracy of screening tools that use questions around consequences of drinking may be particularly affected by local conceptualisations of problematic alcohol use. The findings showed very few adaptations to existing questions, and if researchers had developed their own questionnaires, the questions used were still the same or similar to those in established tools. This may not necessarily be a problem, but further research is needed to understand what this apparent lack of contextual adaptations means for the accuracy of screening tools. No tools have been developed to specifically capture local conceptualisations of alcohol‐related problems in adolescents. A previous review in Eastern Africa noted a lack of use of validated tools [71]. Our review found that almost 40% of studies had used a tool that was validated, although not necessarily for use in African adolescents. Five studies were identified that provided some, albeit very limited and not consistently satisfactorily, evidence of the validity of the AUDIT, TLFB, ASSIST and CRAFFT in our study population. While critical appraisal of these studies was not within the scope of this work, some of these studies fell within the search window of a systematic review on adolescent screening tools which identified no African validation studies above the quality threshold [7]. Future validation studies across the continent and across different settings (rural vs. urban) should explore if existing tools can adequately detect those with alcohol problems, or whether cultural or linguistic adaptations can improve sensitivity. There were no studies that specifically set out to explore cultural applicability of existing questions on alcohol‐related problems.

The findings further showed a limited number of studies that primarily aimed to investigate alcohol use and studies were poorly geographically distributed. The search identified only 58 studies undertaken in the whole of Africa in the last 20 years that used a tool to present data on adolescent harmful drinking, by capturing HED, or screening for disorders. A substantial number of studies were excluded after initial analysis, because they solely presented data on lifetime/current alcohol use. While any alcohol consumption may be of concern, particularly in young adolescents [72], it is unclear if such a measure can adequately identify those in need of an intervention. The authors who presented more in‐depth consumption data undertook their research in only 25 out of 54 African countries. In most countries, only one or two studies were undertaken in the past 20 years, often as part of the GSHS. Around 50% of included studies took place in South‐Africa, a dominance that was also noted in a recent scoping review on alcohol interventions in the region [73]. While this is not something this review set out to explore, this may indicate a large gap in availability of prevalence data on harmful drinking and alcohol‐related problems among adolescents across most of Africa. Moreover, studies that used single items provided only partial insight into consumption patterns. While frequency, quantity and HED items are valid as standalone screening items [7], the WHO advises that surveys of alcohol consumption should contain all three items at a minimum to adequately capture a range of drinking patterns [13]. None of the authors who used single items presented this combination of data and may therefore have under‐identified those with risky drinking patterns. For example, adolescents who drink rarely, but participate in HED when they do, may not be identified as drinking hazardously through a frequency item.

Finally, this review highlighted inconsistencies in thresholds that were applied to determine risk of harm. While different cut‐off scores for binge drinking may represent differences in standard drink sizes in countries, these inconsistencies were also found within countries. Almost all researchers applied adult cut‐off scores to adolescent responses, and no authors stratified their thresholds by age categories. In addition, only a few researchers used lower cut‐off scores for girls than for boys. While this reflects a lack of consensus in the literature about appropriate cut‐off scores, it indicates that already limited studies may have under‐identified alcohol problems in African adolescents, and particularly girls.

To summarise, it is recommended that future research expands its geographical scope across Africa; investigates adolescent alcohol consumption patterns and prevalence of alcohol use disorders in more detail; validates tools, with specific explorations of adolescent‐ and gender‐appropriate cut‐off scores, and the need for cultural adaptations; and explores ways to account for differences in standard drinks across countries and the consumption of locally brewed alcohol.

### 4.1 Study limitations

To the authors' knowledge, this was the first scoping review to map the use of adolescent alcohol consumption, screening and assessment tools in Africa. A significant strength was its rigorous search process and broad inclusion criteria, which led to the inclusion of a large number of studies that used a wide range of tools. However, this study also has some important limitations. First, only studies written in English were included and searches were done only through major scientific databases. Databases specifically indexing African papers (e.g., Africa Wide Information) were not searched. This means that studies published in African languages and in local mediums may not have been identified. Moreover, the inclusion of any study type, without exclusion after critical appraisal, posed challenges related to the quality of reporting. Surveys were very rarely appended and researchers did not always specify the tool that they used. If this was the case, the results section was examined to deduce what type of tool was used. If the results section did not provide a clear indication, the paper was excluded. The authors recognise that this means that the use of certain tools may have been missed and acknowledge this as a limitation to the study. Simultaneously, this highlights an apparent lack of adequate methodological reporting in alcohol research in Africa.

## 5 CONCLUSION

Data on alcohol use and its related problems are needed, not only to monitor the magnitude and trends of alcohol‐related harms, but also to strengthen advocacy and design effective interventions. The findings of this review suggest that data on adolescent alcohol use and disorders across Africa may not be adequately documented due to several reasons, including: a general lack of cross‐sectional studies across many countries; potential unresponsiveness of tools to the local context; inconsistent use of age‐ and gender‐specific cut‐off scores.

## AUTHOR CONTRIBUTIONS

Each author certifies that their contribution to this work meets the standards of the International Committee of Medical Journal Editors. Angela I. Obasi conceived the idea for the project. Search conducted by Eleanor Briegal. *Screening and selection of papers*: Maaike L. Seekles, Eleanor Briegal, Alice M. Biggane with conflicts resolved by Angela I. Obasi. *Extraction of data*: Maaike L. Seeklesand Eleanor Briegal. *Data synthesis and initial drafting of paper*: Maaike L. Seeklesand Eleanor Briegal. Multiple drafts reviewed by all authors: Maaike L. Seekles, Eleanor Briegal, Alice M. Biggane, Angela I. Obasi. Work conducted under supervision of Angela I. Obasi.

## FUNDING INFORMATION

This work was supported by the National Institute for Health Research (Global Health Research Unit on Lung Health and TB in Africa, grant number: 16/136/35) and the UK's Medical Research Council (Public Health Intervention Development Grant, grant number: MR/V032380/1).

## CONFLICT OF INTEREST STATEMENT

The authors declare no conflict of interest.

## Supporting information


**Appendix S1.** Preferred Reporting Items for Systematic Reviews and Meta‐Analyses extension for Scoping Reviews (PRISMA‐ScR) Checklist
**Appendix S2.** MEDLINE search strategy
